# Corticotropin-releasing factor 1 receptor haplotype and cognitive features of major depression

**DOI:** 10.1038/s41398-017-0051-0

**Published:** 2018-01-10

**Authors:** Elena Goetz Davis, Jennifer Keller, Joachim Hallmayer, Heather Ryan Pankow, Greer M. Murphy, Ian H. Gotlib, Alan F. Schatzberg

**Affiliations:** 1 Department of Psychiatry and Behavioral Sciences, Stanford University School of Medicine, Stanford, USA; 2 Department of Psychology, Stanford University, Stanford, USA

## Abstract

Corticotropin-releasing factor signaling through CRF receptor type 1 (CRF_1_) has been shown to contribute to learning and memory function. A haplotype of alleles T-A-T in a set of common polymorphisms in the gene encoding for CRF_1_ (*CRHR1*) has been associated with both depression vulnerability and alterations in cognitive functioning. The present study investigated the relations between the TAT haplotype and specific symptoms of depression, self-reported ruminative behaviors, and neuropsychological performance on a learning and memory task. Participants were adults with major depression with and without psychotic features (*N* = 406). Associations were examined between TAT haplotype and endorsement of depression symptoms from diagnostic interviews, scores on the rumination response scale (RRS), and verbal memory performance on the California Verbal Learning Test-II (CVLT-II). All analyses included depression subtype, age, and sex as covariates; CVLT-II analyses also included evening cortisol levels. Across the entire sample, carriers of more copies of the TAT haplotype reported greater endorsement of the symptom describing difficulty concentrating and making decisions. In separate subsamples, TAT homozygotes had higher rumination scores on the RRS, both brooding and reflection subscales, and more TAT copies were associated with poorer CVLT-II performance in both total learning and free recall trials. These data demonstrate that the *CRHR1* TAT haplotype is associated with cognitive features of depression including difficulty with decision-making, higher rumination, and poorer learning and memory. It will be important in future research to identify the specific molecular mechanisms for CRF_1_ signaling that contribute to depression-related cognitive dysfunction.

## Introduction

Alterations in the corticotropin-releasing factor (CRF) system have been implicated in the pathophysiology of depression, including hyperactivity of the hypothalamic-pituitary-adrenal (HPA) axis stress response and sensitization of amygdala output^1–3^. Signaling through CRF receptor type 1 (CRF_1_) is important for the stress-mediated response of the CRF system^1^, and the expression of this receptor throughout the cortex, hippocampus, and amygdala^4–6^ points to its widespread role beyond the HPA axis. For example, pharmacologic and transgenic manipulations of CRF_1_ signaling in rodent models have been shown to impact hippocampal structure and function^7,8^ and amygdala-mediated learning and memory^9,10^ following stress. In addition, CRF_1_-mediated signaling of CRF in the prefrontal cortex has been shown to underlie working memory performance, independent of stress^11^. Therefore, variation in CRF_1_ signaling may be associated with differences in vulnerability to stress-related disorders, such as depression, as well as differences in cognitive functioning even apart from stress.

In humans, genetic variability in the gene encoding for CRF_1_, *CRHR1*, has been implicated in individual differences in vulnerability to depression and/or severity of depression^12–16^. Specifically, a set of common polymorphisms—rs7209436, rs110402, and rs242924—have been identified as forming a haplotype of alleles T-A-T, respectively, that was originally investigated with respect to its potential protective effect in buffering against increased risk of depression following maltreatment in childhood^12,14^. These gene-by-environment interaction studies focused on the impact of *CRHR1* in sensitizing individuals to an HPA-axis mediated stress cascade, including elevations in cortisol^17–20^. However, results have been mixed: there have been some studies reporting that different types of traumatic stressors^21,22^ or different methods of reporting stressful events in childhood^14^ may change the direction of the effects, and vulnerability in individuals without significant trauma histories does not consistently point to the TAT haplotype^15,23^.

Although this haplotype and other common genetic variants in *CRHR1* have been found to affect stress-system processes including cortisol responses^17,20^, the actual specific effects of the TAT polymorphisms on *CRHR1* functioning have not been elucidated and likely extend beyond the HPA axis. Previous findings from our research group have found *CRHR1* genotype, including rs110402 within the TAT haplotype, is associated with increased likelihood of psychotic features in severe major depressive disorder, even when controlling for cortisol levels as a proxy of HPA axis dysregulation^16^. Genetic variation in *CRHR1* has been observed and/or posited to affect cognitive processes, include memory, rumination, and decision-making^24–26^, as well as the formation of emotional memories^14^. Such findings suggest that the TAT haplotype plays a role in influencing cognitive functioning in depression.

The present study was designed to investigate whether the TAT haplotype is associated with cognitive features in currently depressed individuals. We examined depression symptom endorsement, self-reported ruminative behavior, and neuropsychological performance on a test of learning and memory in individuals diagnosed with major depressive disorder (MDD) as a function of the number of TAT haplotype copies. Our analyses included important covariates: age, sex, and depression subtype (with or without psychotic features). Because of previous equivocal findings, we sought to obtain convergent evidence of the role of TAT across both self-report and objective measures of cognition. In our first set of analyses, we also took an unbiased approach considering the link between the haplotype and all symptoms of depression, to examine the importance of the TAT haplotype in explaining heterogeneity within MDD including the domain of cognition as well as other symptoms. In addition, in contrast with several previous studies that focused on gene-by-environment (GxE) interactions with childhood trauma, we sought to investigate whether there was a main effect of the TAT haplotype on maladaptive cognition, drawing from our finding linking *CRHR1* variation with psychotic features of MDD^16^. Our community sample of adults with depression was not selected to be a traumatized cohort. We obtained information about histories of post-traumatic stress symptoms and diagnoses on the majority of participants, and investigated associations between TAT haplotype and cognition in the entire sample as well as the non-traumatized subsample. In addition, to further investigate whether variation in *CRHR1* at these loci relates to cognition in a way that is separable from a traumatic reaction and stress-related cascade, we sought to link genotype with neuropsychological performance even after controlling for individuals’ levels of cortisol secretion. Finally, because of known associations between *CRHR1* variation and vulnerability to alcohol use disorders^27–29^ as well as sex differences in the links between *CRHR1* and psychopathology^21,28,30^, we obtained information about substance use histories in our sample and investigated potential moderation of our findings by sex.

## Materials and methods

### Participants

Data for the following specific analyses were obtained from subsets of adult participants who were recruited from the community between 2000 and 2013 to complete treatment, behavioral, and/or neuroimaging research studies on depression. These samples were combined for analysis through the Biocollaborative Database at the Stanford Mood Disorders Center, which is a collaborative data-sharing effort designed to allow for interrogation of specific genetic variation in cohorts including depressed patients. All participants included in this study were administered a standardized diagnostic interview—either the Structured Clinical Interview for DSM-IV^31^ (SCID; *N* = 395) or the Mini International Neuropsychiatric Interview^32^ (M.I.N.I.; *N* = 11)—and were required to meet DSM-IV criteria for MDD either with (PMD) or without psychotic features (NPMD). Participants also were required to have no history of psychotic disorder and no substance abuse or dependence in the past 6 months. Lifetime substance use disorder incidence was low in this sample (14/406; 3.4%). To reduce heterogeneity in the sample and potential for population stratification since ancestry markers were not available^12^, all participants included in these analyses were Caucasian and non-Hispanic. All procedures were approved by the Stanford Institutional Review Board, and all participants provided informed consent.

### Procedures

The entire sample was included in the analysis of depression symptom endorsement, and two non-overlapping subsamples were used in the rumination and neuropsychological performance analyses, all described below.

#### Depression symptoms

This sample consisted of 406 adults with current MDD (373 NPMD, 33 PMD). Symptom-level data from MDD modules were used in the analyses, recoded to reflect either threshold endorsement of the symptom or absent/subthreshold symptom (binary); missing data was 0.5%. Logistic regression was used to examine the effect of TAT haplotype count on symptom endorsement across the nine MDD symptoms, first controlling for diagnostic group (NPMD vs. PMD), participant age, and participant sex. We also examined sex as a potential moderator of the TAT effect in a separate analysis. To control for multiple comparisons, *α* < 0.01 was used to determine statistical significance, and significant results were followed up with tests of symptom specifiers, if applicable. In addition, the majority of participants completed the PTSD module of the SCID (386/406) and 80% reported no lifetime threshold or subthreshold PTSD. Main analyses were repeated in this subsample (*N* = 307). The Hamilton Depression Rating Scale-17 item version (HDRS-17) was used as an index of disorder severity in *N* = 362 participants with available data^33,34^.

#### Rumination scores

Rumination data were available for 185 NPMD participants who completed the Rumination Response Scale^35^ (RRS), a 22-item self-report measure assessing the frequency of engaging in different forms of ruminative thinking. The brooding subscale captures a passive and negatively-focused thought process that is posited to be maladaptive and associated with depression both concurrently and prospectively, whereas the reflection subscale captures purposeful efforts at problem-solving that may be linked to negative affect and depression symptoms in the short term, but are adaptive over time^36^. RRS brooding and reflection subscale scores were examined using analyses of covariance (ANCOVAs) to determine whether they differed by TAT haplotype count across the entire sample, with age and sex included as covariates. Finally, we examined sex as a potential moderator of the TAT effect.

#### Neuropsychological performance

Neuropsychological performance data were available for 55 participants (25 NPMD, 30 PMD) who completed the California Verbal Learning Test-2^nd^ Edition^37^ (CVLT-II), a list-learning task in which participants hear 16 words and are given multiple trials to learn them. Verbal memory performance is indexed by total number of words learned, as well as by short-delay recall, long-delay recall, and recognition performance. Additional performance measures include strategy type used while learning (semantic, serial, or subject clustering) and number of repetitions within learning trials. All participants also completed overnight hourly blood draws at the Stanford Hospital Clinical and Translational Research Unit to obtain levels of cortisol and were included in the sample only if they were not currently taking hormonal replacement therapy or oral contraceptives. Hierarchical linear regression analyses were used to predict verbal memory performance as a function of TAT haplotype count, controlling for diagnostic group (NPMD vs. PMD), age, sex, and mean evening cortisol levels. Cortisol levels were included as a covariate because they were previously found to influence cognitive function in this sample, and to be elevated in psychotic compared to nonpsychotic MDD^16,38,39^; we sought to determine effects of the haplotype apart from any relation to psychotic features or cortisol. Finally, we examined sex as a potential moderator.

### Genotyping

DNA was derived from either saliva or blood. Saliva samples were collected via Oragene kits and DNA was purified and extracted according to standardized protocols (dnagenotek.com). DNA was extracted from EDTA-treated whole blood using the Gentra Puregene kit (Qiagen, Valencia, CA). All DNA samples were genotyped for a set of 19 common single nucleotide polymorphisms (SNPs) across the *CRHR1* gene using iPlex reagents on a MassArray System (Agena Bioscience, Inc., San Diego, CA, USA) following the manufacturer’s protocol under standard conditions or using a Taqman platform from Applied Biosystems^16^. The *CRHR1* TAT haplotype consists of the T allele (vs. C) for rs7209436, the A allele (vs. G) for rs110402, and the T allele (vs. C) for rs242924^14^. Haplotypes were confirmed using PHASE software with >90% certainty^40^, and the number of copies of the TAT haplotype were coded (0, 1, 2).

### Cortisol determination

During an overnight stay, at 16 h on Day 0 (baseline), an intravenous line was started in one arm and 8 cc of blood were drawn every hour from 1800 to 0900 h the following morning in order to assay cortisol and adrenocorticotropic hormone (ACTH) levels. Subjects were required to lie in bed 15 min prior to each blood sampling. Plasma was immediately separated from whole blood by centrifuge and then stored at −70 °C before assay. Cortisol concentrations were measured using the Access Immunoassay System (Beckman Coulter, Chaska, MN). The sensitivity is 0.4 µg/dL (11 nmol/L) and the precision within assays is 6.4–7.9%. Cortisol assays were conducted by the Brigham & Women’s Hospital, General Clinical Research Laboratory in Boston.

Because of the natural diurnal rhythm of the cortisol slopes, the 15-h blood collection period was divided into two phases based on the apparent nadir: the evening level from 1800 to 0100 h and the morning level from 1 to 9 h. These epochs correspond to the natural descending and ascending slopes of the cortisol, and the parent study from which these samples were drawn includes a figure displaying the curve of cortisol levels over this time period^16^. Based on previous findings indexing significant variation among depressed patients captured by the evening epoch^38,41^, we used the mean levels of cortisol from this time period as the covariate in our regression analyses. Evening cortisol level mean values were 4.1 +/− 2.0 µg/dL for the NPMD group and 5.0 +/− 2.9 µg/dL for the PMD group, and morning cortisol values were 9.9 +/− 3.0 µg/dL for the NPMD group and 10.0 +/− 5.1 µg/dL for the PMD group.

## Results

### Genotype results

All three SNPs that comprise the TAT haplotype were found to be in Hardy-Weinberg equilibrium in each of the three samples analyzed, *p*s > = .99. Haplotype counts within each sample are presented in Table 1.

**Table 1:** *CRHR1* TAT haplotype distributions in the study samples (CVLT-II: California Verbal Learning Test-2^nd^ Edition)

Analysis sample	TAT count: 0 copies	TAT count: 1 copy	TAT count: 2 copies	Total
Depression symptoms	135	176	95	406
Rumination scores	57	86	42	185
CVLT-II performance	19	26	10	55

### Depression symptom endorsement

The majority of participants in this analysis were female (*N* = 268 female, 66%), and they were on average 43.0 +/− 12.1 years of age (range 18–74 years). Control analyses in subsamples with available data revealed that neither MDD severity (HDRS-17 score), F(2, 360) = 0.36, *p* = .70, nor PMD status, *χ*^2^(2) = 4.81, *p* = .09, differed by TAT haplotype group.

**Fig. 1:**
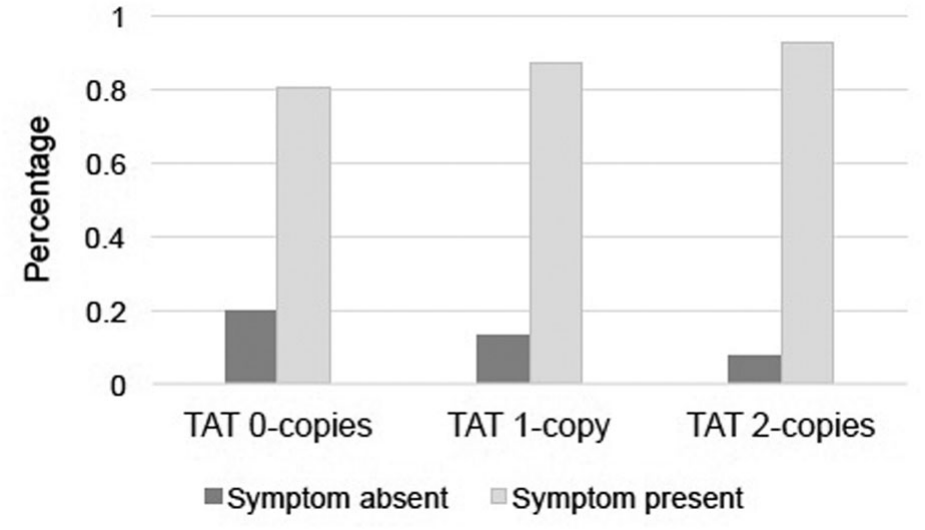
Percentage of patients endorsing depression symptom “diminished ability to think or concentrate, or indecisiveness,” by *CRHR1* TAT haplotype group Individuals were more likely to endorse the symptom with increasing numbers of TAT copies, controlling for age, sex, and diagnostic group (major depressive disorder with vs. without psychotic features), (*χ*^2^(1) = 7.02, *p* = .008)

Evidence was found for differential endorsement of only one symptom of depression as a function of TAT haplotype count that met our a priori statistical threshold (see Table 2). For the symptom “diminished ability to think or concentrate, or indecisiveness,” TAT count predicted symptom endorsement beyond the effects of the covariates, *χ*^2^(1) = 7.02, *p* = .008, *B* = 0.53, Exp(B) = 1.70 for the TAT effect, indicating greater levels of symptom endorsement with more TAT copies, model Nagelkerke *R*^2^ = 0.056 (see Fig. 1). As a follow-up, endorsement of the specifier symptoms “diminished ability to think” vs. “indecisiveness” were investigated separately in regression models for participants with available data (*N* = 388); only “indecisiveness” was found to significantly differ by TAT count, *χ*^2^(1) = 13.82, *p* < .001 (“difficulty concentrating” *p* = .49). Separate models that examined sex as moderator of the TAT effect found no evidence of an interaction: *p*s > .15 for the models predicting the concentration symptom and specifiers, and *p*s > .22 for models of the other symptoms.

**Table 2:** Results of logistic regression models with *CRHR1* TAT haplotype count predicting symptom endorsement (binarized), controlling for participant age, sex, and diagnostic group (major depressive disorder with vs. without psychotic features)

Symptom	*N*	Percent endorsed (%)	Step 1: age, sex, and diagnostic group	Step 2: TAT haplotype count
Depressed mood	406	90.4	*χ*^2^(3) = 1.88, *p* = .60	*χ*^2^(1) = 6.38, *p* = .012
Loss of interest	406	94.1	*χ*^2^(3) = 0.71, *p* = .87	*χ*^2^(1) = 3.78, *p* = .052
Change in appetite	403	55.1	*χ*^2^(3) = 3.04, *p* = .39	*χ*^2^(1) = 1.12, *p* = .29
Change in sleep	401	75.1	*χ*^2^(3) = 5.69, *p* = .13	*χ*^2^(1) = 0.05, *p* = .82
Psychomotor agitation/retardation	402	46.8	*χ*^2^(3) = 19.48, *p* < .001	*χ*^2^(1) = 3.59, *p* = .058
			(Group: *p* < .001)	
Loss of energy	406	89.4	*χ*^2^(3) = 0.83, *p* = .84	*χ*^2^(1) = 0.06, *p* = .80
Feelings of guilt/worthlessness	402	80.3	*χ*^2^(3) = 2.04, *p* = .56	*χ*^2^(1) < 0.001, *p* = .99
Difficulty concentrating/ indecisiveness	404	86.1	*χ*^2^(3) = 5.74, *p* = .13	*χ*^2^(1) = 7.02, *p* = .008
Suicidality	405	36.0	*χ*^2^(3) = 15.36, *p* = .002	*χ*^2^(1) = 1.27, *p* = .26
			(Group: *p* = .002)

Analyses were repeated in the non-PTSD subsample (*N* = 307). Lifetime PTSD endorsement did not differ by TAT group, *χ*^2^(2) = 2.29, *p* = .32. TAT haplotype count significantly predicted depressed mood, *χ*^2^(1) = 6.38, *p* = .012, loss of interest, *χ*^2^(1) = 5.35, *p* = .021, and difficulty concentrating/making decisions, *χ*^2^(1) = 8.72, *p* = .003, although only the concentration symptom met our stringent statistical threshold. Similarly, as above, the specifier analysis yielded a significant effect only for indecisiveness, *χ*^2^(1) = 15.53, *p* < .001, and not for difficulty concentrating, *p* = .23.

### Rumination scores

**Fig. 2:**
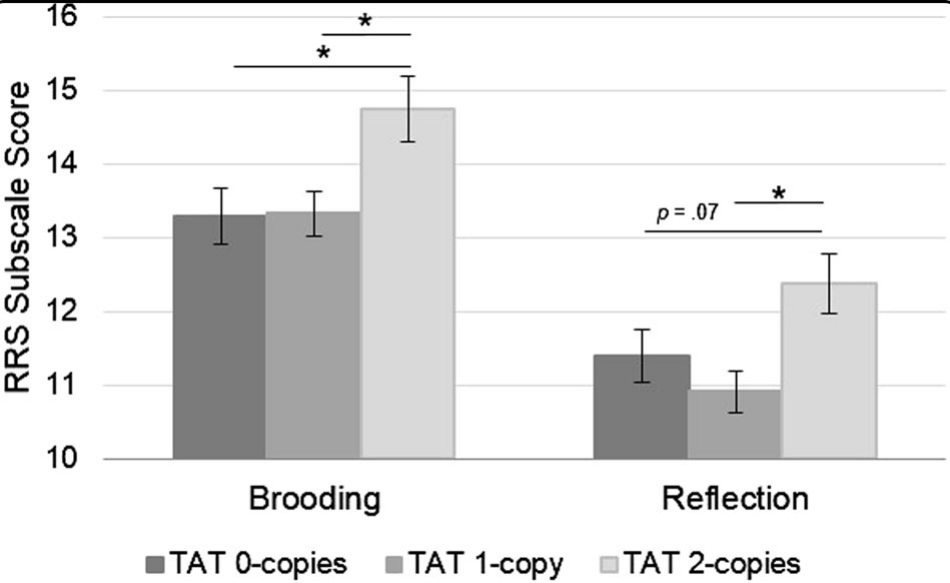
**Rumination self-report ratings are highest among TAT homozygotes.** Brooding and reflection subscale scores from the Rumination Response Scale (RRS) significantly differ across TAT haplotype group, controlling for age and sex (plot displays estimated marginal means and standard errors, * *p* < .05)

Participants who completed the RRS (*N* = 185) were majority female (66%) and averaged 41.5 +/− 11.2 years of age. Age was inversely correlated with both RRS subscales (*r* = −.15, *p* = .049 for brooding; *r* = −.17, *p* = .23 for reflection), but age did not differ by TAT group (F(2, 182) = 1.01, *p* = .37). ANCOVAs conducted with age and sex as a covariates indicated that levels of both brooding and reflection differed as a function of TAT count, F(2, 180) = 4.20, *p* = .017, and F(2, 180) = 4.36, *p* = .014, respectively (Fig. 2). Pairwise comparisons showed TAT homozygotes had greater brooding rumination than did both 1- and 0-copy carriers (*p* = .008 and .012 respectively). TAT homozygotes also reported greater reflection than did the 1-copy carrier group (*p* = .004), but did not differ significantly from the 0-copy group, although their mean was higher at a trend level, *p* = .07. There was no evidence of sex as a significant moderator of these TAT effects: F(2, 178) = .85, *p* = .43 and F(2, 178) = .16, *p* = .86 for the interaction term in the brooding and reflection models, respectively. Error variances of RRS subscales did not differ across groups, *p*s > .05, and subscales were normally distributed overall and for each group, skewness < |0.4|, kurtosis < |0.7|.

### Neuropsychological performance

Participants who completed the CVLT-II (*N* = 55) were majority female (60%) and 42.4 +/− 14.5 years old. Proportion of females and average age did not differ between the NPMD and PMD diagnostic groups (*χ*^2^(1) = 1.22, *p* = .27; F(1, 53) = .33, *p* = .57; respectively), and the groups also did not differ by years of education (PMD 15.1 +/− 2.8; NPMD 15.4 +/− 1.8; F(1, 53) = 0.27, *p* = .61). Mean evening cortisol level was not associated significantly with either TAT haplotype, F(2, 49) = 1.10, *p* = .34, or diagnostic group, F(1, 49) = 0.79, *p* = .38, nor with the interaction of haplotype and diagnostic group, F(2, 49) = 0.03, *p* = .97.

Results of the regression analyses are presented in Table 3. Age, sex, diagnostic group, and cortisol significantly predicted CVLT-II total learning over time and both short-delay and long-delay free recall. Adding the TAT haplotype to each model significantly increased the prediction of learning and memory performance. For the learning trials, TAT haplotype explained an additional 14.1% of the variance above and beyond the other variables (Δ*R*^2^ F(1, 49) = 10.55, *p* = .002). TAT haplotype contributed similarly for short-delay and long-delay free recall, with TAT accounting for an additional 9.7–14.0% of the variance (see Table 3). Both higher age and an increasing TAT count were negatively associated with total number of words learned, whereas the effect of diagnostic group indicates that non-psychotic depression status was associated with a greater number of words recalled. The regression models did not significantly predict recognition performance, F(5, 49) = 1.43, *p* = .23 or the number of repetitions, F(5, 49) = 2.14, *p* = .077. Furthermore, the regression models did not predict the use of semantic (*R*^2^ = 18.1%, F(5, 49) = 2.17 *p* = .073) or serial (*R*^2^ = 19.3%, F(5, 49) = 2.34 *p* = .055) clustering strategies, though the overall model predicted subjective clustering strategy (*R*^2^ = 24.5%, F(5, 49) = 3.17 *p* = .015). However, there was no effect of TAT count on subjective clustering strategy use, Δ*R*^2^*p* = .29. Finally, there was no evidence that sex moderated these effects: all *p*s > .45.

**Table 3:** Results of hierarchical regression models with *CRHR1* TAT haplotype count predicting cognitive performance on the California Verbal Learning Test-2nd Edition (CVLT-II), controlling for participant age, sex, diagnostic group (major depressive disorder with vs. without psychotic features), and evening cortisol levels

CVLT-II variable	Full model: age, sex, diagnostic group, cortisol, and TAT haplotype count	Step 1: age, sex, diagnostic group, and cortisol	Step 2: TAT haplotype count	Significant predictors in full model
Total learning over trials 1–5	*R*^2^ = 34.6%	*R*^2^ = 20.5%	Δ*R*^2^ = 14.1%	Age *t* = −3.33, *p* = .002
	F(5, 49) = 5.19,	F(4, 50) = 3.23,	F(1, 49) = 10.55,	TAT *t* = −3.25, *p* = .002
	*p* = .001	*p* = .020	*p* = .002	
Short-delay, free recall	*R*^2^ = 30.6%,	*R*^2^ = 16.6%,	Δ*R*^2^ = 14.0%,	Group *t* = 2.15, *p* = .037
	F(5, 49) = 4.32,	F(4, 50) = 2.49,	F(1, 49) = 9.91,	TAT *t* = −3.18, *p* = .003
	*p* = .002	*p* = .055	*p* = .003	
Long-delay, free recall	*R*^2^ = 27.2%,	*R*^2^ = 17.5%,	Δ*R*^2^ = 9.7%,	Group *t* = 2.62, *p* = .012
	F(5, 49) = 3.66,	F(4, 50) = 2.66,	F(1, 49) = 6.52,	TAT *t* = −2.55, *p* = .014
	*p* = .007	*p* = .043	*p* = .014	

Full model statistics and *R*^2^ are given for each step; *N* = 55 included in analysis

In examining the subsample without PTSD histories (*N* = 42), the amount of variance accounted for by the TAT haplotype remained similar when predicting CVLT-II performance (ranging from 9.7–12.5%). Overall models predicting total learning and short-delay and long-delay free recall including all predictors were significant, with TAT haplotype adding significantly to the models: Total learning over five trials (Δ*R*^2^ = 9.7%, F(1, 36) = 5.85, *p* = .021); short-delay free recall (Δ*R*^2^ = 12.5%, F(1, 36) = 6.70, *p* = .014); long-delay free recall (Δ*R*^2^ = 10.6%, F(1, 36) = 5.78, *p* = .022). The non-PTSD group had even stronger findings that were consistent with the overall sample indicating that increasing TAT count was negatively associated with total number of words learned and recalled.

## Discussion

In a large cohort of depressed adults, we identified an association between TAT haplotype and more frequent endorsement of the MDD symptom indicating cognitive difficulties^31^. Within subsamples of the larger cohort, we also identified associations between TAT haplotype and higher rumination, both brooding and reflection types, and diminished learning and memory performance on a standard neuropsychological test of verbal memory. These results are consistent across multiple domains in showing that carriers of the TAT haplotype demonstrate maladaptive cognitive functioning associated with depression.

Our finding indicating that the TAT haplotype may function as a risk factor for poorer cognitive functioning in a depressed sample is somewhat counter to seminal association studies that identify a protective role of the TAT haplotype, although those studies focused on protection from developing depression following early life stress^12,14^. In our samples, the majority of depressed individuals had no lifetime diagnoses of even subthreshold PTSD, indicating a group without clinically significant adverse reactions to trauma. The direction of the association we identified is consistent with studies assessing community cohort samples with minimal evidence of trauma^15,23^ where findings pointed to TAT carriers being at risk for greater depressive symptom scores, and for developing depression in the absence of childhood adversity^28^. Our results show that despite no TAT group differences in overall severity of depression, as assessed by total HRSD-17 score, our findings point specifically to haplotype-associated differences in endorsement of cognitive symptoms of depression. Specifically, both the specific depressive symptom of indecisiveness as well as rumination are associated with the TAT haplotype. Genetic variation in *CRHR1* has been linked to impaired decision-making in the Iowa Gambling Task^26^, and the TAT tagging SNP rs110402 has been associated with psychotic symptoms in a mixed cohort of depressed and healthy individuals^16^, providing converging evidence of its important role in cognitive functioning.

Our results point to individual differences in *CRHR1* encoding for a cognitive profile with clinical relevance for the functioning of depressed patients and approaches to intervention for this disorder. Genetic variation in *CRHR1* has been shown to be relevant for antidepressant treatment response and also to capture heterogeneity in MDD symptom presentation^42^. Evidence of poorer cognitive performance can inform treatment selection for NPMD patients; indeed, there have been reports of preferential responses to specific antidepressants such as vortioxetine or duloxetine, the latter particularly in the elderly^43–45^. The relevance of the TAT haplotype for decisions concerning intervention is highlighted in a recent study in which interpersonal psychotherapy was found to improve depressive symptoms, increase social adjustment, and decrease perceived stress at post-treatment, but only among women with 0 copies of the *CRHR1* TAT haplotype^46^. These data and our results are consistent with a differential susceptibility model in which individuals with 0 TAT copies are responsive both to stressful and to nurturing environments^47^; indeed, investigators have referred to the major alleles at rs242924 and rs110402 the “stress-responsive” alleles^24^. Previous studies have documented associations between TAT and both greater rumination^48,49^ and poorer working memory^24^, but only when in the context of investigating GxE interaction models that incorporate significant life stressors such as childhood adversity or maternal depression. In the present study we found that in the absence of specific stressful or traumatic environmental exposures, TAT carriers have relatively poorer cognitive functioning.

In this large sample of depressed adults, we identified a direct link between TAT haplotype and cognitive functioning independent of age and, importantly, found that the link with neuropsychological performance was independent of cortisol levels. Glucocorticoid signaling has known effects on cognition^50^; in particular, higher cortisol levels have been associated with poorer cognitive performance on the CVLT-II^51^. In the present study, however, TAT haplotype predicted approximately 9–14% of the variance in CVLT-II scores beyond the effects of cortisol. In addition, the TAT haplotype has been associated with dysregulated diurnal and stress-responsive cortisol patterns^17,18,20^, but all in the context of childhood maltreatment. Despite these links, in this study and our previous report^16^, *CRHR1* genotype did not predict evening cortisol levels. The effects of CRF signaling through CRF_1_ can be distinct from glucocorticoid levels, as was shown in Alzheimer’s disease, a disorder characterized by profound cognitive impairment^52^, and also can be independent of the HPA axis^53^.

The functional impact of the TAT haplotype or genetic variation in proximal SNPs of *CRHR1* is currently unknown, and our findings cannot elucidate the mechanism by which TAT is related to cognition. Intriguing evidence that implicates this system includes the neuroprotective effects of CRF_1_ signaling, even those that can be counter to the negative effects of cortisol^54–56^. Our results show an association with diverse yet related forms of disrupted cognition, and it is unclear if the common underlying process is related to attention, regulation, memory, or more broadly, to CRF_1_-mediated plasticity or neuroprotection within hippocampal or cortical neurons. Some investigators have speculated that the TAT haplotype plays a role in emotional memory^14^, particularly negative memory consolidation due to the role of CRF_1_ in fear conditioning^9,10^. It is possible that highly emotional or unregulated processing of negative memories manifests as rumination and also interferes with encoding of new information or with decision-making in daily life. Although we cannot link these distal phenotypes to the biology of *CRHR1* genetic variants, our findings point to a role of CRF_1_ in these depression-related cognitive processes.

Despite the interest and importance of the present results, there are several limitations that should be considered. First, although the overall sample of depressed individuals was reasonably sized (*N* > 400), the subsamples for the rumination and CVLT-II analyzes were notably smaller. Thus, a larger total sample would be helpful in replicating these results, particularly the neuropsychological performance analyses capturing an objective measure of cognitive performance. Second, although not the focus of our study, more information about the trauma histories in our sample would have been helpful in reconciling our results with findings from other studies of TAT. Our sample was a community cohort of adults diagnosed with MDD and, in the majority of individuals, we had information about PTSD history that was sufficient to characterize our sample as relatively low trauma; however, it will be important in future research to include scales assessing history, degree, and type of trauma. In addition, there is evidence that estrogens can influence CRF signaling and CRF_1_ regulation^57,58^ and medications that affect estrogen such as hormone replacement therapy can influence cortisol levels^59^. We did not have information to address the potential confound of differing estrogen levels for our participants in the symptom and rumination analyses, although individuals taking hormone replacement therapy or oral contraceptives were excluded from the sample in the neuropsychological performance analysis. Finally, this study focused on a depressed cohort and a larger scale study of cognitive performance in healthy controls would help address whether there is similar cognitive risk in even non-disordered individuals, as well as individuals with other forms of psychopathology influenced by CRF_1_ variation such as substance use disorders.

The TAT-cognition association may function as a trade-off in adaptiveness, depending on one’s stressful or traumatic life circumstances and overall cognitive functioning. We show here that individuals without significant early adversity manifest more cognitive problems associated with depression if they are TAT haplotype homozygotes. These findings underscore the need to gain a better understanding of how genetic variation in *CRHR1* can have functional effects that lead to phenotypes of relevance to psychopathology, with potential implications for understanding individual differences in the manifestation of MDD and targets for more effective treatment of this disorder.
